# Distinct Longitudinal Changes in Immunoglobulin G N-Glycosylation Associate with Therapy Response in Chronic Inflammatory Diseases

**DOI:** 10.3390/ijms23158473

**Published:** 2022-07-30

**Authors:** Jerko Štambuk, Frano Vučković, Siniša Habazin, Maja Hanić, Mislav Novokmet, Susanna Nikolaus, Florian Tran, Stefan Schreiber, Andre Franke, Philip Rosenstiel, Gordan Lauc, Konrad Aden, Marija Pezer

**Affiliations:** 1 Genos Glycoscience Research Laboratory, 10000 Zagreb, Croatia; jstambuk@genos.hr (J.Š.); fvuckovic@genos.hr (F.V.); shabazin@genos.hr (S.H.); mhanic@genos.hr (M.H.); mnovokmet@genos.hr (M.N.); glauc@genos.hr (G.L.); 2 Department of Internal Medicine, University Hospital Schleswig-Holstein, 24105 Kiel, Germany; s.nikolaus@mucosa.de (S.N.); f.tran@ikmb.uni-kiel.de (F.T.); s.schreiber@mucosa.de (S.S.); a.franke@mucosa.de (A.F.); p.rosenstiel@mucosa.de (P.R.); k.aden@ikmb.uni-kiel.de (K.A.); 3 Institute of Clinical Molecular Biology, Christian-Albrechts-University Kiel, 24118 Kiel, Germany; 4 Faculty of Pharmacy and Biochemistry, The University of Zagreb, 10000 Zagreb, Croatia

**Keywords:** chronic inflammatory diseases, inflammatory bowel disease, IgG glycosylation, response, personalized medicine, autoimmune diseases

## Abstract

Immunosuppressants and biologicals are widely used therapeutics for various chronic inflammatory diseases (CID). To gain more detailed insight into their downstream effects, we examined their impact on serum immunoglobulin G (IgG) glycosylation. We analyzed IgG subclass-specific fragment crystallizable (Fc) N-glycosylation in patients suffering from various CID using the LC-MS approach. Firstly, we compared IgG Fc N-glycosylation between 128 CID patients and 204 healthy controls. Our results replicated previously observed CID-related decrease in IgG Fc galactosylation (adjusted *p*-value range 1.70 × 10^−2^–5.95 × 10^−22^) and sialylation (adjusted *p*-value range 1.85 × 10^−2^–1.71 × 10^−18^). Secondly, to assess changes in IgG Fc N-glycosylation associated with therapy and remission status, we compared 139 CID patients receiving either azathioprine, infliximab, or vedolizumab therapy. We observed an increase in IgG Fc galactosylation (adjusted *p*-value range 1.98 × 10^−2^–1.30 × 10^−15^) and sialylation (adjusted *p*-value range 3.28 × 10^−6^–4.34 × 10^−18^) during the treatment. Furthermore, patients who reached remission displayed increased Fc galactosylation levels (*p*-value range 2.25 × 10^−2^–5.44 × 10^−3^) in comparison to patients with active disease. In conclusion, the alterations in IgG Fc glycosylation and the fact these changes are even more pronounced in patients who achieved remission, suggest modulation of IgG inflammatory potential associated with CID therapy.

## 1. Introduction

Chronic inflammatory diseases (CID) affect various organs such as the intestinal tract, skin, or joints and represent a common disease group of the immune system with rising incidence in Western industrialized countries. CID arise at the interplay of genetic risk, individual lifestyle, and environmental factors. Chronicity, long-term debilitating consequences due to anatomical destruction of the affected organ systems (mucosal atrophy, joint destruction, scar formation, intestinal failure), and the lack of causative treatments are among the factors that explain the high individual burden of disease.

Since there is no curative therapy available for CID, treatments are mostly focused on reducing symptoms and inflammation, and have to be given lifelong [1]. The current mainstay in the therapy of moderate to severe Crohn’s disease (CD) and ulcerative colitis (UC) is the use of targeted therapies (anti-TNF, anti-IL-12/23, anti-α4β7 integrin), so-called biologics, to intercept the chronic perpetuation of mucosal inflammation. However, the long-term efficacy of biologics is restricted due to primary and secondary loss of response in a large proportion of patients [2]. Whereas primary non-response describes a refractory state to a given drug at treatment induction, secondary loss of response develops in patients over time after having initially undergone therapy-induced amelioration of bowel inflammation. Despite growing evidence of host and microbial factors involved in the development of primary non-response to, e.g., anti-TNF treatment, a thorough understanding of the underlying mechanisms of developing secondary loss of response is scarce [3,4,5]. With respect to anti-TNF therapy, the development of neutralizing anti-drug antibodies against anti-TNF has shown to substantially contribute to secondary loss of response, leading to proactive therapeutic drug monitoring as a key concept in anti-TNF therapy across several CID [6,7]. However, with regard to different treatment modalities, such as anti-α4β7 integrin or anti-IL12/23 treatment, the understanding of underlying molecular mechanisms contributing to primary or secondary loss of response is limited [8].

The majority of cell surface and secreted proteins are N-glycosylated, while the absence of N-glycosylation results in embryonic death in murine models [9]. Glycosylation affects protein structural and functional features and is thus involved in the regulation of numerous biological processes [10,11]. There is no genetic template for glycosylation. Instead, genetic and environmental factors influence glycan synthesis in complex biosynthetic pathways. Consequently, the structure of N-glycans depends, among other things, on the expression and activity of enzymes and the availability of their substrates. Changes in glycan levels can be seen in different physiological and pathological states. Numerous diseases such as inflammatory diseases, cancers, and autoimmune diseases display extensive changes in protein N-glycosylation patterns [12,13].

Immunoglobulin G (IgG) is the most abundant antibody in plasma and is a key molecule of the humoral immune response, linking innate and adaptive immunity through its multiple roles. IgG can be divided into four subclasses: IgG1, IgG2, IgG3, and IgG4 with different affinities towards Fc and other receptors, thus distinctive effector functions [14]. Each IgG molecule contains a conserved N-glycosylation site on a fragment crystallizable (Fc); and a sporadic N-glycosylation site in the Fab region resulting from somatic hypermutation events [14]. The most complex IgG Fc glycan contains 12 monosaccharide units and represents a biantennary digalactosylated and monosialylated structure with bisecting β(1,4) *N*-acetylglucosamine (GlcNAc) and an α(1,6) fucose attached to the core GlcNAc. The remaining IgG glycans correspond to this structure with the lack of one or more sugar units.

Changes in Fc glycans alter IgG conformation and interactions with different receptors, which define specific downstream immune responses [15,16]. Sweeping changes in IgG inflammatory functions can be observed with various IgG glycan patterns [17,18]. For example, low levels of galactosylation and core fucosylation are associated with higher inflammatory potential, while the presence of core fucose and sialic acids fosters anti-inflammatory functions of IgG [19,20]. Pro-inflammatory IgG glycan patterns—the most striking effect being a lowered proportion of galactosylated structures—were observed in a wide range of chronic inflammatory diseases (CID), including various autoimmune diseases such as Crohn’s disease (CD), ulcerative colitis (UC), rheumatoid arthritis (RA), and systemic lupus erythematosus (SLE) [20,21,22,23]. Moreover, IgG glycan patterns also associate with UC, CD, and SLE severity and differ between CD and UC patients [22,24]. There are several mechanisms for how deficiency of terminal galactoses on IgG can act in a pro-inflammatory manner. Proposed mechanisms include activation of complement via the alternative pathway, or via the lectin pathway after binding to mannose-binding lectin [25,26]. However, the exact mechanism of action, through which the lack of terminal galactoses activates pro-inflammatory IgG properties, is still a subject of debate [20,27]. When it comes to the effects of high IgG Fc galactosylation levels, immune complexes can more effectively activate the anti-inflammatory cascade, through binding to the FcγRIIB receptor, if galactoses are present on IgG Fc glycan [28]. In a similar way, a high level of IgG galactosylation is required for immune complexes to inhibit the pro-inflammatory activity of the C5a complement component [29]. On the other hand, a lack of galactose can also act in a pro-inflammatory way. The presence of galactose on IgG Fc increases complement-dependent cytotoxicity (CDC) through the classical pathway of complement activation, while elevated IgG galactosylation levels also activate antibody-dependent cellular cytotoxicity (ADCC) through enhanced binding of galactosylated IgG to activating FcγRs [30,31]. 

The aims of this study were (1) to replicate patterns in IgG glycosylation previously reported in patients suffering from chronic inflammatory diseases compared to control subjects, (2) to examine the impact of targeted therapies on IgG Fc glycan patterns over time, and (3) to identify potential patterns of IgG Fc glycosylation that are associated with clinical response to targeted therapies in CID.

## 2. Results

The first goal of this study was to replicate previously reported changes in IgG glycosylation patterns observed in CID patients. For that reason, we compared IgG Fc glycosylation of CID patients with healthy controls. Secondly, we examined the impact of immunosuppressive and biological therapies on IgG Fc glycosylation in patients with CID. To answer this question, we analyzed IgG Fc N-glycan profiles in CID patients on azathioprine and biological therapies: anti-TNF and anti-α4β7 integrin monoclonal antibodies—infliximab and vedolizumab, respectively. Patients originated from three independent cohorts and were followed up for 104 (Cohort 1), 30 (Cohort 2), or 14 (Cohort 3) weeks. Thirdly, we compared the extent of modification of IgG glycan profiles between patients in remission and patients with active disease to evaluate the potential of IgG glycans as predictors of disease activity.

### 2.1. IgG Fc Galactosylation and Sialylation Are Lower in Chronic Inflammatory Diseases Patients Compared to Controls

**Figure 1:**
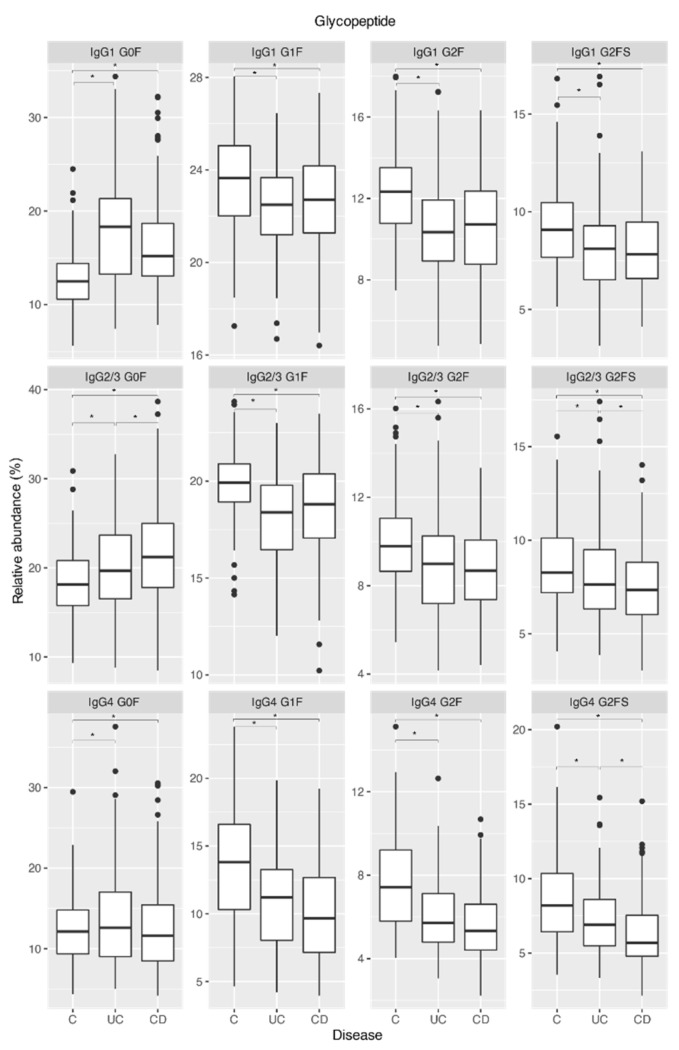
IgG Fc-glycan patterns in controls, patients suffering from ulcerous colitis, and patients suffering from Crohn’s disease (Cohort 1 at baseline) for all IgG subclasses. Each box represents the 25th to 75th percentiles (interquartile range—IQR). Lines inside boxes stand for the median. The whiskers are the lowest and highest values within boxes ±1.5 × the IQR. Dots are outliers (>1.5 × IQR). Significant differences (*p* < 0.05) are marked with an asterisk *. Normalized size effect and *p*-values are given in Table S1. C—controls; UC—ulcerous colitis; CD—Crohn’s disease.

In order to replicate previously reported changes in IgG glycosylation in chronic inflammatory diseases, we compared CID patients to a control group. At baseline (prior to initiation of therapy), patients from Cohort 1 suffering from UC (*n* = 12) and CD (*n* = 32) displayed considerable shifts in IgG Fc glycan profiles compared to controls (*n* = 204; Figure 1). The nature of observed changes in IgG Fc glycans with the disease was similar for all measured IgG subclasses (Table S1). Agalactosylated glycan species (represented by G0F glycoform) increased, while mono- and di-galactosylated glycans (represented by G1F and G2F glycoforms, respectively) decreased in all subclasses. Changes in IgG4 galactosylation traits showed smaller effect sizes compared to the other two subclass clusters, and the difference in IgG4 G0F abundance in CD patients compared to controls did not reach statistical significance. IgG Fc sialylation levels (G2FS) showed a significant decrease with comparable effect size across all subclasses in both UC and CD patients. Therefore, overall galactosylation and sialylation levels decreased in CID patients compared to the control cohort.

Differences in IgG Fc glycan profiles were also observed between the two disease entities: Compared to UC patients’ glycan profiles, CD patients in the IgG2/3 subclass showed an increase in agalactosylation levels (G0F) and decreased presence of terminal sialic acid (G2FS). CD patients also showed a decrease in terminal sialic acid (G2FS) in the IgG4 subclass compared to UC patients (Figure 1, Table S1).

In Cohort 1, similar differences in IgG glycosylation, compared to controls, were observed when, besides UC (*n* = 12) and CD (*n* = 32), patients suffering from other chronic inflammatory diseases were taken into account. Namely, patients with several types of arthritis (psoriatic arthritis—PsA, *n* = 22; seropositive rheumatoid arthritis—RA+, *n* = 37; seronegative rheumatoid arthritis—RA−, *n* = 19; and systemic lupus erythematosus—SLE, *n* = 6) also showed lower IgG Fc galactosylation and sialylation when compared to controls (Tables S2–S4).

### 2.2. IgG Fc Galactosylation and Sialylation Levels Increase during Therapy

**Figure 2:**
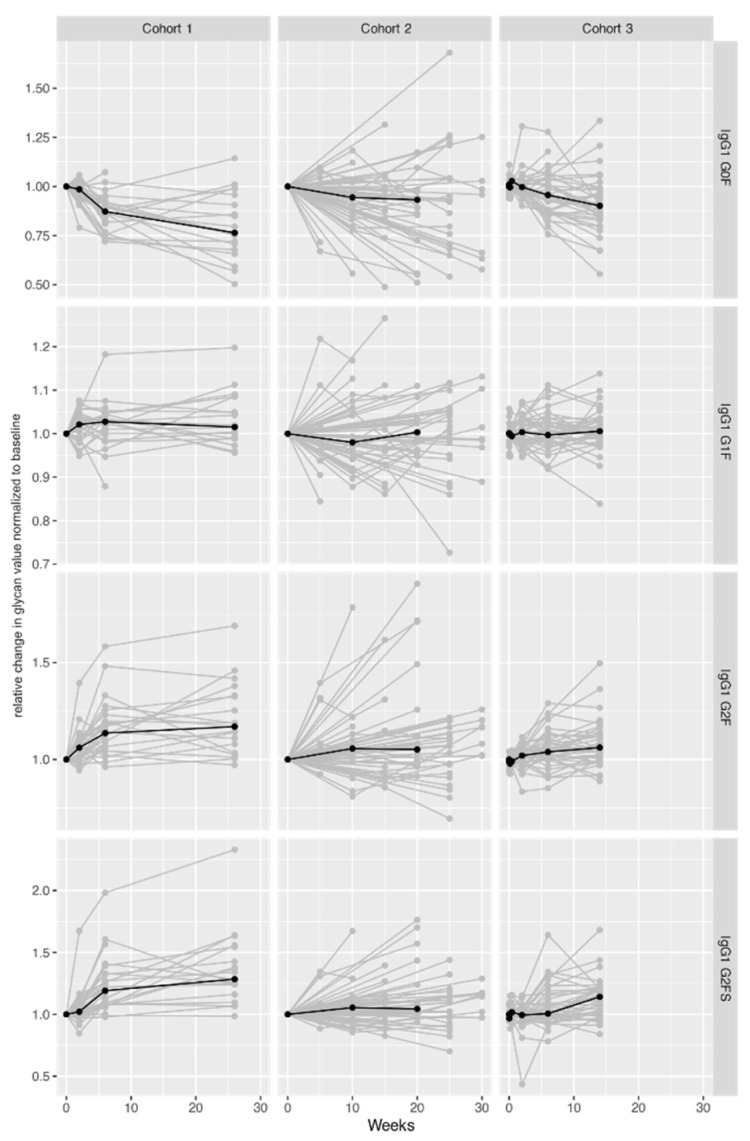
Changes in IgG1 Fc glycan profiles during treatment in three cohorts of UC and CD patients. Median glycan values for each time point are bolded. Y-axis: relative change in glycan value normalized to baseline (1st timepoint); X-axis: duration of follow-up (weeks). Results of meta-analysis are shown in Table 1, while the results of statistical analysis for individual cohorts are shown in Table S5.

Having shown that CID patients display a significantly altered IgG glycosylation profile in comparison to controls, we next aimed to understand whether a targeted biologic therapy dynamically affects IgG Fc glycosylation patterns over time. The statistical analysis has shown that during therapy (Cohort 1—26 weeks, Cohort 2—30 weeks, and Cohort 3—14 weeks) IgG glycan profiles of 34 UC and 113 CD patients changed over time regardless of their clinical response (Figure 2, Table S5). 

A significant change was detected for most of the observed 12 glycan traits, with all three cohorts showing very similar patterns of change. In all cohorts, galactosylation and sialylation levels increased (decrease in G0F accompanied by an increase in G2F and G2FS) for IgG1 and IgG2/3. For the IgG4 subclass, this pattern was observed only in Cohort 1. In the other two cohorts, only some changes reached statistical significance: a decrease in G0F in Cohort 2, and a decrease in G0F accompanied by an increase in G2FS in Cohort 3.

Meta-analysis of all three cohorts confirmed the change of IgG Fc glycan pattern over time on therapy. IgG Fc galactosylation and sialylation levels of all three IgG subclasses showed a significant increase in patients on therapy. The increase in galactosylation was represented by a decrease in G0F accompanied by an increase in G2F structures, while an increase in G2FS structure represented increased sialylation (Table 1).

To further verify whether longitudinal changes in IgG glycosylation patterns are a unifying feature of targeted therapies in all chronic inflammatory disorders, we validated our findings on patients with psoriatic arthritis (PsA, *n* = 22), seronegative rheumatoid arthritis (RA-, *n* = 19), seropositive rheumatoid arthritis (RA+, *n* = 37), ankylosing spondylitis (AS, *n* = 17), UC (*n* = 12), and CD (*n* = 32) that were monitored after therapy initiation (Cohort 1) (Figure S1). Within 26 weeks of therapy, CD patients and all arthritis patients showed a significant change in IgG1 and IgG2/3 Fc glycosylation (Tables S6 and S7). In addition, several Fc glycans of the IgG4 subclass significantly changed with therapy for AS, CD, RA-, and RA+ patients (Table S8). Interestingly, in ulcerative colitis patients, Fc glycosylation patterns did not significantly change in any IgG subclass.

### 2.3. Pronounced IgG Fc Galactosylation Associates with Clinical Remission in CID Therapy

**Figure 3:**
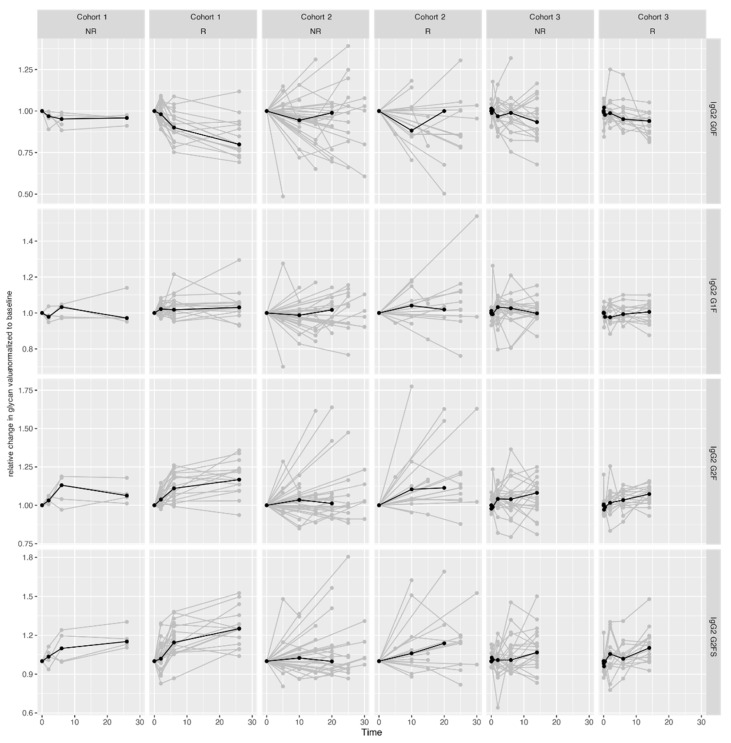
Changes in IgG2/3 Fc glycan profiles during treatment in CID (UC and CD) patients that entered remission (R) vs. patients with active disease (NR) in three cohorts. Median glycan values for each time point are bolded. Y-axis: relative change in glycan value normalized to baseline (1st timepoint); X-axis: duration of follow-up (weeks). Associations of IgG Fc glycan profiles with disease activity during treatment, for each patient cohort separately, are shown in Table S9, while the results of meta-analysis of all three cohorts are presented in Table 2.

Having shown that targeted therapy changes the IgG Fc glycosylation patterns across all CID, and irrespectively of the therapeutic outcome, we next aimed to identify specific IgG glycosylation patterns that are indicative of therapy response in CID. For that purpose, we further analyzed glycosylation patterns in 34 UC and 113 CD patients based on their clinical remission status. We observed that patients in Cohort 2 who reached clinical remission displayed a starker increase in IgG2/3 subclass digalactosylation (G2F) since therapy initiation compared to patients with active disease. A similar, but even more pronounced change was independently validated in Cohort 3, where patients in remission showed a starker increase in digalactosylation and sialylation accompanied by a starker decrease in agalactosylation (G0F) of the IgG2/3 subclass (Figure 3, Figures S2 and S3, Table S9).

In Cohort 1, there were no significant differences in the rate at which IgG Fc glycan patterns change between patients that achieved remission and patients with active disease. A meta-analysis of all three cohorts revealed two substantial differences in the rate of IgG Fc glycan profiles changes between patients who did vs. patients who did not achieve remission: patients who entered remission displayed a more pronounced increase in digalactosylation of IgG2/3 and a more pronounced decrease in agalactosylation of IgG4 (Table 2 and Table S9).

We also found that IgG glycosylation levels measured at baseline do not provide information on future disease activity, i.e., response to therapy. Namely, there is no statistically significant difference between patients who enter vs. patients who do not enter remission in their IgG Fc glycan profiles measured at therapy initiation (Table S10).

## 3. Discussion

In this study, we analyzed subclass-specific IgG Fc glycosylation profiles in CID patients treated with immunosuppressive and biological therapies. We replicated previously published alterations of IgG glycan profiles in UC, CD, arthritis, and SLE patients when compared to controls [24,32]. Furthermore, in UC, CD, and arthritis patients on azathioprine and biological therapy, we observed changes in IgG Fc glycosylation which reflect a less inflammatory IgG glycopattern: an increase in the proportion of galactosylated and sialylated structures. Moreover, UC and CD patients undergoing remission showed a more pronounced increase in anti-inflammatory IgG Fc glycan levels when compared to non-remission patients.

The most pronounced change we saw in IgG Fc glycan patterns, which occurred in CID patients at baseline, was a decrease in IgG galactosylation levels. Such a shift in IgG glycan profiles towards lower IgG galactosylation levels, which is generally considered a pro-inflammatory property, can be observed in various inflammatory and autoimmune diseases and conditions [20,22,33]. There are several proposed mechanisms through which the absence of galactose on IgG glycans can act in a pro-inflammatory manner; however, these are still subject to debate [26,28,34]. Besides changes in galactosylation levels, we also detected a decrease in sialylation in all IgG subclasses of CID patients. Sialylation of IgG glycans is considered a switch between pro- and anti-inflammatory activity of the IgG molecule through modulation of binding to activating FcγRs, lectin (type II) receptors, and the C1q complement component [34,35]. However, these reports remain controversial and exact mechanisms remain elusive [20,36]. In general, a decrease in IgG galactosylation and sialylation when compared to control subjects is considered a potential modulator of the immune activation threshold, reflecting a general pro-inflammatory environment in CID patients, which was observed in numerous studies (reviewed in [20,37]).

The effect of immunosuppressive and biological therapy on IgG Fc glycan patterns in CID patients was examined by monitoring changes in Fc glycan levels during treatment. The most pronounced change was in the galactosylation level, which significantly increased during treatment in all cohorts. Interestingly, the direction of this change was irrespective of the remission status of patients. We observed the same trends in various arthritis patients on anti-TNF therapy, indicating that this change is neither disease nor drug specific. Similar shifts in glycan patterns were observed in several published longitudinal studies showing recovery of serum IgG galactosylation levels in rheumatoid and psoriatic arthritis patients treated with immunosuppressive and biological therapy [38,39,40,41]. Recently published studies demonstrated changes in glycan profiles of IgG or total plasma proteins in CD and UC patients and attributed glycan profiles to medication or disease course [24,32,42].

There are several lines of evidence showing that the observed glycosylation profiles could be attributed to the suppressed systemic inflammation resulting from biological or azathioprine therapy. Although the mechanisms of changes in IgG Fc glycosylation following CID therapy are still unclear, the majority of treatments included in this study show a profound impact on the fate of B cell lineage. Azathioprine in high doses suppresses B cell activity and differentiation [43,44]. Of the used biologicals, anti-TNF therapy was found to restore the number of circulating B cells in patients on therapy close to levels observed in healthy controls [45]. Furthermore, it has been observed that anti-TNF treatment changed the expression of glycosyltransferases in bovine synovitis [46]. On the other hand, blocking of the α4β7 integrin pathway with vedolizumab did not affect the level of plasma cells [8,47].

The link between IgG glycosylation and inflammation is demonstrated by dynamic changes in IgG galactosylation levels in female RA patients during and after pregnancy. During pregnancy, RA patients experience hormone-related remission of disease and an increase in IgG galactosylation to near-normal levels. However, in the postpartum period, the disease relapses accompanied by a decrease in IgG galactosylation to the levels before pregnancy [48,49].

Apart from changes in galactosylation, we have also detected an increase in sialylation levels in patients on therapy, which is thought to have a protective effect against inflammation. This observation matches IgG glycan changes on native self-targeting autoantibody complexes observed in RA patients showing the reduced inflammatory potential of IgG [50]. Therefore, observed changes of IgG Fc glycan profiles during therapy and regardless of patients’ clinical response status suggest a reduced inflammatory potential of IgG, likely as a consequence of suppressed therapy-associated systemic inflammation [20].

We also showed changes in IgG glycan profiles of UC and CD patients in remission induced by azathioprine and biological drugs. Although this change was not detected in Cohort 1, the metanalysis of all three cohorts together showed that patients in remission displayed a more pronounced increase in IgG2/3 digalactosylation levels and a more pronounced decrease in IgG4 agalactosylation levels when compared to patients with active disease. Our findings are supported by a smaller previous study of 19 CD patients monitored before and two weeks after initiation of anti-TNF therapy, where a significant increase in galactosylation was found in responders even at baseline [51]. The observed increase in galactosylation suggests a further reversal of IgG glycosylation towards a healthy non-inflammatory IgG glycan profile in patients in remission. Based on the presented data, longitudinal monitoring of IgG Fc glycosylation can serve as an additional indicator of the remission status.

Since the early prediction of therapy response and disease outcome is an essential therapeutic goal, we assessed the predictive power of the IgG glycosylation pattern at baseline. Based on IgG Fc glycan levels at baseline, we were not able to segregate patients based on their future remission status.

We acknowledge several limitations of our study: The three included patient cohorts are not completely matched in terms of therapy and nosology. However, these are longitudinal cohorts that were analyzed independently of each other, and the initial status of a single cohort was used as its baseline. Furthermore, we did not analyze every specific diagnosis and therapeutic modality separately, as a small number of patients in these groups and an increased number of tests would have led to inadequate statistical power to detect relevant effects.

In conclusion, we replicated differences in IgG glycosylation patterns between CID patients and control subjects. Moreover, we further expanded the knowledge about the CID therapy effect on IgG glycome composition, which implies that therapy-driven shifts in IgG Fc glycan patterns are neither disease nor drug specific. Finally, we discovered differences in the rate of IgG Fc glycome change between patients who eventually entered remission vs. patients with active CID. Altogether, our findings show extensive modulation of IgG inflammatory potential driven by the applied medications.

## 4. Materials and Methods

### 4.1. Patient Samples

Three longitudinal cohorts of CID patients were used all naïve to therapy (Table S11). Patients were fully informed as to the purpose of the study and signed a written consent in accordance with the Helsinki declaration. The ethics committee of the Christian-Albrechts-Universität zu Kiel approved the study (A 124/14).

Cohort 1 comprised 146 patients with CD, UC, SLE, or various arthritic diseases: psoriatic arthritis (PsA), seronegative rheumatoid arthritis (RA-), seropositive rheumatoid arthritis (RA+), ankylosing spondylitis (AS). Patients were on anti-TNF therapy (infliximab), and blood samples were collected at multiple time points after initiation of therapy (up to 104 weeks). For easier comparison with other cohorts in this study, only the four earliest time points (baseline, 2, 6, and 26 weeks) were used for statistical analysis. AS samples were not used in baseline comparison due to missing baseline timepoints. SLE patients were not longitudinally analyzed through their observation period due to a small patient number.

Two hundred and four healthy participants served as controls for Cohort 1 patients at baseline.

Cohort 2 comprised 61 patients treated with azathioprine and anti-TNF. Blood samples were collected at baseline and two of the four additional time points during follow-up: 2 weeks, 6 weeks, 14 weeks, and 30 weeks.

Cohort 3 included 42 patients who were prescribed infliximab or vedolizumab. Blood samples were collected by venipuncture at baseline, 2 weeks, 6 weeks, and 14 weeks after commencement of therapy.

### 4.2. Immunoglobulin G Isolation

IgG was isolated from plasma samples using protein G affinity chromatography as described previously [52]. In short, 100 μL plasma from each sample was centrifuged at 1620× *g* and filtered through a 0.45 μm pore filter plate to remove aggregated lipids. Filtered plasma was then diluted eight times with an in-house prepared 1× phosphate-buffered saline (PBS) and loaded onto a 96-well protein G monolithic plate (BIA Separations, Ajdovščina, Slovenia). Each well of the monolithic plate was washed three times with 2 mL of 1× PBS and IgG was eluted from protein G using 1 mL of 0.1 M formic acid (Merck, Darmstadt, Germany). The eluate was immediately neutralized by adding 170 μL of 1 M ammonium bicarbonate (Across Organics, Pittsburgh, PA, USA). IgG concentrations were measured using NanoDrop 8000 (Thermo Fisher Scientific, Waltham, MA, USA).

### 4.3. IgG Trypsin Digestion and Solid-Phase Extraction of Glycopeptides

IgG was digested with trypsin and the obtained glycopeptides were purified as described before with slight changes [53]. In brief, 0.1 μg of sequencing grade trypsin (Promega, Fitchburg, WI, USA) was added to 40 μL (on average ~20 μg) of isolated IgG and incubated overnight at 37 °C. The digest was then diluted ten times using 0.1 % (*v*/*v*) trifluoracetic acid (TFA) and loaded to C18 ec sorbent (Macherey-Nagel, Düren, Germany). The samples were washed three times with 200 μL of 0.1% TFA and eluted from the phase with 20% liquid chromatography-mass spectrometry (LC-MS) grade acetonitrile (Honeywell, Morris Plains, NJ, USA). The eluted glycopeptides were vacuum-dried and redissolved in 80 μL of ultrapure water.

### 4.4. Liquid Chromatography-Mass Spectrometry Analysis of IgG Fc Glycopeptides

IgG Fc glycopeptides were separated on an Acquity M-class chromatographic system (Waters, Milford, MA, USA), which was coupled to a Compact mass spectrometer (Bruker, Bremen, Germany) using a CaptiveSpray source equipped with a nanoBooster. Glycopeptides (6 μL) were loaded onto PepMap 100 C8 (5 mm × 300 μm i.d.; Thermo Fisher Scientific, Waltham, MA, USA) in a mobile phase A (0.1 % TFA) at a flow rate of 40 μL/min. IgG subclasses were separated on a C18 analytical column (150 mm × 100 µm i.d., 100 Å; Advanced Materials Technology, Wilmington, DE, USA) in a 3.5-min-long gradient from 16% to 25% of mobile phase B (80% can in 20% solvent A) at a flow rate of 1 μL/min. The column temperature was maintained at 30 °C. Acetonitrile vapors were introduced directly into the source using a nanoBooster to increase the ionization of glycopeptides. Mass spectra were recorded using an *m*/*z* range of 600–1900 with 0.5 Hz and averaging two sequential scans. The collision and quadrupole energies were set to 4 eV, while transfer time and pre-pulse storage were set to 110 μs and 10 μs, respectively. The nanoACQUITY UPLC system was controlled by MassLynx software version 4.1 (Waters), while the mass spectrometer was controlled by HyStar software version 4.1.2 (Bruker).

### 4.5. Data Processing

Data were extracted using LacyTools v1.0.1 software [54]. The peak intensity of exact mass was adjusted to a predefined value for each of the separated subclasses for spectra alignment. The three most intensive glycoforms (G0F, G1F, and G2F) were used for alignment. The time window to search for maximal peak intensity was set to 55 s and the mass window was 0.2 *m*/*z*. Only samples with a minimum of five analytes per sample with signal-to-noise (S/N) higher than nine were aligned. In Caucasian populations, the IgG2 and IgG3 glycopeptides have the same peptide sequence, resulting in the ions of the same *m*/*z*. Therefore, these two subclasses cannot be separated by LC-MS [55]. Fc glycopeptides of the four subclasses were therefore chromatographically separated into only three clusters: IgG1, IgG2/3, and IgG4. In the time window of 30 s around the defined time, spectra were summed, and a sum spectrum was created for each of the separated IgG subclasses. Three calibrants with S/N larger than nine were used for the calibration of sum spectra with a calibration window of 0.2 Da. Integration of areas containing 99% of the isotopic pattern was performed, followed by summing of doubly and triply charged species originating from a single analyte. Signals corresponding to agalactosylated (G0F), monogalactosylated (G1F), digalactosylated (G2F), and sialylated (G2FS) Fc glycans carrying core fucose were extracted.

### 4.6. Statistical Analysis

Normalization and batch correction were performed on the LC-MS glycopeptide data to remove experimental variation from the measurements. Normalization by total area was performed to make measurements across samples comparable. Prior to the batch correction, normalized glycan measurements were log-transformed because of the right-skewness of their distributions and the multiplicative nature of batch effects. The batch correction was performed on log-transformed measurements using the ComBat method (R package sva), where the plate designation was modeled as a batch covariate for each sample. Estimated batch effects were subtracted from log-transformed measurements to correct measurements for experimental noise.

Longitudinal analysis of samples through their observation period was performed by implementing a linear mixed-effects model where time was modeled both as a fixed effect and random slope, the interaction between time and therapy response was modeled as a fixed effect, while individual sample ID was modeled as a random intercept. Analyses were first performed for each cohort separately and then combined using the inverse-variance weighted meta-analysis approach (R package metafor). Prior to the analyses, glycan variables were all transformed to a standard normal distribution by the inverse transformation of ranks to normality (R package “GenABEL”; function rntransform). Using rank transformed variables makes estimated effects of different glycans comparable as these will have the same standardized variance. The Benjamini–Hochberg procedure was used to control the false discovery rate (FDR) at the specified level of 0.05. Data were analyzed and visualized using the R programming language (version 3.5.2).

## Figures and Tables

**Table 1:** Changes in IgG Fc glycan profiles during therapy in three cohorts of UC and CD patients: meta-analysis (Cohort 1–26 weeks, Cohort 2–30 weeks, and Cohort 3–14 weeks on therapy). *p*-values were adjusted for multiple testing and considered significant if <0.05 (bold). Effect: model coefficient (slope) representing the weekly change of a glycan trait (expressed in standard deviation units). Changes in IgG1 Fc glycan patterns during treatment for individual cohorts are visualized in Figure 2 and results of statistical analysis for individual cohorts are shown in Table S5.

IgG Subclass	Glycan	Effect	Standard Error	*p*-Value	Adjusted *p*-Value
IgG1	G0F	**−2.93 × 10^−2^**	4.00 × 10^−3^	2.41 × 10^−13^	**7.24 × 10^−13^**
	G1F	7.98 × 10^−4^	3.41 × 10^−3^	8.15 × 10^−1^	8.15 × 10^−1^
	G2F	**2.25 × 10^−2^**	3.12 × 10^−3^	5.64 × 10^−13^	**1.35 × 10^−12^**
	G2FS1	**2.89 × 10^−2^**	3.23 × 10^−3^	3.62 × 10^−19^	**4.34 × 10^−18^**
IgG2/3	G0F	**−2.10 × 10^−2^**	3.06 × 10^−3^	7.74 × 10^−12^	**1.55 × 10^−11^**
	G1F	4.25 × 10^−3^	3.33 × 10^−3^	2.02 × 10^−1^	2.20 × 10^−1^
	G2F	**2.09 × 10^−2^**	2.56 × 10^−3^	3.25 × 10^−16^	**1.30 × 10^−15^**
	G2FS1	**2.16 × 10^−2^**	2.48 × 10^−3^	2.76 × 10^−18^	**1.66 × 10^−17^**
IgG4	G0F	**−1.95 × 10^−2^**	3.13 × 10^−3^	5.03 × 10^−10^	**8.62 × 10^−10^**
	G1F	**−6.43 × 10^−3^**	2.68 × 10^−3^	1.65 × 10^−2^	**1.98 × 10^−2^**
	G2F	**1.19 × 10^−2^**	3.59 × 10^−3^	9.35 × 10^−4^	**1.25 × 10^−3^**
	G2FS1	**1.48 × 10^−2^**	3.12 × 10^−3^	2.19 × 10^−6^	**3.28 × 10^−6^**

**Table 2:** Associations between the rate of change of IgG Fc glycan profiles and disease activity during UC and CD treatment—meta-analysis of three cohorts (Cohort 1–26 weeks, Cohort 2–30 weeks, and Cohort 3–14 weeks). Effect: the difference between two model coefficients (slopes), where each coefficient represents a group-specific weekly change of glycan trait (expressed in standard deviation units). *p*-values were adjusted for multiple testing and considered significant if <0.05 (bold). Changes in IgG glycan patterns with time on therapy in patients with active vs. inactive disease are visualized in Figure 3, Figures S2 and S3, and results of statistical analysis for individual cohorts are shown in Table S9.

IgG Subclass	Glycan	Effect	Standard Error	*p*-Value	Adjusted *p*-Value
IgG1	G0F	−1.45 × 10^−2^	7.61 × 10^−3^	5.61 × 10^−2^	1.35 × 10^−1^
	G1F	−3.86 × 10^−3^	6.50 × 10^−3^	5.52 × 10^−1^	6.02 × 10^−1^
	G2F	8.27 × 10^−3^	6.25 × 10^−3^	1.85 × 10^−1^	3.18 × 10^−1^
	G2FS1	7.89 × 10^−3^	6.38 × 10^−3^	2.16 × 10^−1^	3.24 × 10^−1^
IgG2/3	G0F	−1.36 × 10^−2^	5.58 × 10^−3^	1.50 × 10^−2^	6.00 × 10^−2^
	G1F	1.24 × 10^−2^	5.75 × 10^−3^	3.08 × 10^−2^	9.23 × 10^−2^
	G2F	**1.81 × 10^−2^**	5.16 × 10^−3^	4.54 × 10^−4^	**5.44 × 10^−3^**
	G2FS1	8.14 × 10^−3^	5.34 × 10^−3^	1.28 × 10^−1^	2.55 × 10^−1^
IgG4	G0F	**−1.69 × 10^−2^**	5.85 × 10^−3^	3.75 × 10^−3^	**2.25 × 10^−2^**
	G1F	−5.56 × 10^−3^	5.75 × 10^−3^	3.34 × 10^−1^	4.00 × 10^−1^
	G2F	6.82 × 10^−4^	7.43 × 10^−3^	9.27 × 10^−1^	9.27 × 10^−1^
	G2FS1	7.06 × 10^−3^	6.61 × 10^−3^	2.85 × 10^−1^	3.80 × 10^−1^

## Data Availability

The data that support the findings of this study are available from authors upon reasonable request.

## Supplementary Materials

The following supporting information can be downloaded at: https://www.mdpi.com/article/10.3390/ijms23158473/s1.

## Abbreviations

ADCC	antibody-dependent cellular cytotoxicity
AS	ankylosing spondylitis
CD	Crohn’s disease
CDC	complement-dependent cytotoxicity
CID	chronic inflammatory diseases
Fc	fragment crystallizable
GlcNAc	*N*-acetylglucosamine
IBD	inflammatory bowel disease
IgG	immunoglobulin G
G	galactose
F	fucose
S	*N*-acetylneuraminic acid (sialic acid)
IL-6	interleukin 6
LC-MS	liquid chromatography-mass spectrometry
PBS	phosphate buffered saline
PsA	psoriatic arthritis
RA	rheumatoid arthritis
SLE	systemic lupus erythematosus
S/N	signal-to-noise
TNF	tumor necrosis factor
UC	ulcerative colitis
