# Supplementary material for: Distinct Longitudinal Changes in Immunoglobulin G N-Glycosylation Associate with Therapy Response in Chronic Inflammatory Diseases
Source: Int J Mol Sci. 2022 Jul 30;23(15):8473. doi: 10.3390/ijms23158473 (PMC9368836; doi:10.3390/ijms23158473)
Supplement: Supplementary file 1 [file ijms-23-08473-s001.zip › ijms-1808971-supplementary.pdf]

Supplementary Data

Table S1: Baseline associations of IgG Fc glycan profiles with ulcerative colitis (UC) and Crohn's disease (CD). Effect: the difference of a glycan trait (expressed in standard deviation units) between disease (Cohort 1) and control groups and between UC and CD patients. *p*-values were adjusted for multiple testing and considered significant if <0.05 (bold).

IgG subclass	Glycan	Disease	Effect	Standard error	*p*-value	Adjusted *p*-value
**IgG1**	**G0F**	CD	**1.01**	0.10	1.68E-23	**2.98E-22**
**IgG1**	**G1F**	CD	**-0.54**	0.10	4.44E-07	**7.27E-07**
**IgG1**	**G2F**	CD	**-0.95**	0.10	5.29E-21	**3.81E-20**
**IgG1**	**G2FS**	CD	**-0.84**	0.10	4.17E-16	**1.50E-15**
**IgG1**	**G0F**	UC	**1.08**	0.10	6.54E-25	**2.35E-23**
**IgG1**	**G1F**	UC	**-0.72**	0.11	4.99E-10	**1.06E-09**
**IgG1**	**G2F**	UC	**-0.92**	0.10	6.80E-18	**3.06E-17**
**IgG1**	**G2FS**	UC	**-0.71**	0.11	5.60E-11	**1.34E-10**
**IgG1**	**G0F**	UC vs. CD	-0.19	0.11	8.90E-02	1.07E-01
**IgG1**	**G1F**	UC vs. CD	0.22	0.12	5.92E-02	7.59E-02
**IgG1**	**G2F**	UC vs. CD	0.00	0.11	9.71E-01	9.71E-01
**IgG1**	**G2FS**	UC vs. CD	-0.06	0.11	6.17E-01	6.35E-01
**IgG2/3**	**G0F**	CD	**0.95**	0.10	2.08E-20	**1.25E-19**
**IgG2/3**	**G1F**	CD	**-0.69**	0.11	2.20E-10	**4.94E-10**
**IgG2/3**	**G2F**	CD	**-0.99**	0.09	2.48E-23	**2.98E-22**
**IgG2/3**	**G2FS**	CD	**-0.86**	0.10	2.82E-17	**1.13E-16**
**IgG2/3**	**G0F**	UC	**0.61**	0.11	2.73E-08	**5.46E-08**
**IgG2/3**	**G1F**	UC	**-0.85**	0.11	2.44E-13	**7.33E-13**
**IgG2/3**	**G2F**	UC	**-0.79**	0.10	1.09E-13	**3.58E-13**
**IgG2/3**	**G2FS**	UC	**-0.58**	0.11	8.39E-08	**1.51E-07**
**IgG2/3**	**G0F**	UC vs. CD	**0.37**	0.11	1.20E-03	**1.80E-03**
**IgG2/3**	**G1F**	UC vs. CD	0.19	0.12	1.15E-01	1.33E-01
**IgG2/3**	**G2F**	UC vs. CD	-0.20	0.11	6.11E-02	7.59E-02
**IgG2/3**	**G2FS**	UC vs. CD	**-0.27**	0.11	1.39E-02	**1.92E-02**
**IgG4**	**G0F**	CD	0.11	0.11	2.97E-01	3.15E-01
**IgG4**	**G1F**	CD	**-0.73**	0.10	6.29E-13	**1.62E-12**
**IgG4**	**G2F**	CD	**-1.03**	0.10	9.75E-23	**8.78E-22**
**IgG4**	**G2FS**	CD	**-0.96**	0.10	1.67E-19	**8.57E-19**
**IgG4**	**G0F**	UC	**0.34**	0.11	2.09E-03	**3.00E-03**
**IgG4**	**G1F**	UC	**-0.60**	0.11	6.69E-08	**1.27E-07**
**IgG4**	**G2F**	UC	**-0.85**	0.11	3.11E-13	**8.61E-13**
**IgG4**	**G2FS**	UC	**-0.61**	0.12	2.22E-07	**3.81E-07**
**IgG4**	**G0F**	UC vs. CD	-0.15	0.11	1.76E-01	1.93E-01
**IgG4**	**G1F**	UC vs. CD	-0.16	0.12	1.77E-01	1.93E-01
**IgG4**	**G2F**	UC vs. CD	-0.24	0.12	4.19E-02	5.58E-02
**IgG4**	**G2FS**	UC vs. CD	**-0.41**	0.12	6.50E-04	**1.02E-03**

Table S2: Baseline associations of IgG1 Fc glycans with various chronic inflammatory diseases from the Cohort 1 *p*-values were adjusted for multiple testing and considered significant if <0.05 (bold). Effect: the difference of a glycan trait (expressed in standard deviation units) between patient and control groups. Diseases: psoriatic arthritis (PsA), seronegative rheumatoid arthritis (RA-), seropositive rheumatoid arthritis (RA+), ankylosing spondylitis (AS), ulcerative colitis (UC), Crohn's disease (CD), systemic lupus erythematosus (SLE).

Disease	Glycan	Effect	Standard error	*p*-value	Adjusted *p*-value
**CD**	**G0F**	**1.01**	0.10	1.68E-23	**5.95E-22**
**CD**	**G1F**	**-0.54**	0.10	4.44E-07	**7.62E-07**
**CD**	**G2F**	**-0.95**	0.10	5.29E-21	**7.62E-20**
**CD**	**G2FS**	**-0.84**	0.10	4.17E-16	**2.73E-15**
**PsA**	**G0F**	**0.68**	0.09	1.75E-12	**6.62E-12**
**PsA**	**G1F**	**-0.22**	0.10	3.14E-02	**3.33E-02**
**PsA**	**G2F**	**-0.61**	0.09	1.23E-11	**4.01E-11**
**PsA**	**G2FS**	**-0.57**	0.09	2.57E-10	**6.60E-10**
**RA-**	**G0F**	**0.87**	0.14	3.88E-09	**8.72E-09**
**RA-**	**G1F**	0.01	0.17	9.44E-01	9.44E-01
**RA-**	**G2F**	**-0.73**	0.13	4.75E-08	**9.77E-08**
**RA-**	**G2FS**	**-0.62**	0.14	1.09E-05	**1.57E-05**
**RA+**	**G0F**	**0.93**	0.11	2.72E-15	**1.51E-14**
**RA+**	**G1F**	**-0.30**	0.13	2.56E-02	**2.75E-02**
**RA+**	**G2F**	**-0.86**	0.10	3.51E-16	**2.53E-15**
**RA+**	**G2FS**	**-0.74**	0.11	5.80E-11	**1.67E-10**
**SLE**	**G0F**	**0.89**	0.13	3.10E-11	**9.71E-11**
**SLE**	**G1F**	**-0.56**	0.14	6.37E-05	**8.82E-05**
**SLE**	**G2F**	**-1.03**	0.12	4.97E-16	**2.98E-15**
**SLE**	**G2FS**	**-0.80**	0.12	2.75E-10	**6.83E-10**
**UC**	**G0F**	**1.08**	0.10	6.54E-25	**4.71E-23**
**UC**	**G1F**	**-0.72**	0.11	4.99E-10	**1.20E-09**
**UC**	**G2F**	**-0.92**	0.10	6.80E-18	**6.12E-17**
**UC**	**G2FS**	**-0.71**	0.11	5.60E-11	**1.67E-10**

Table S3: Baseline associations of IgG2/3 Fc glycans with various chronic inflammatory diseases from the Cohort 1. *p*-values were adjusted for multiple testing and considered significant if <0.05 (bold). Effect: the difference of a glycan trait (expressed in standard deviation units) between patient and control groups. Diseases: psoriatic arthritis (PsA), seronegative rheumatoid arthritis (RA-), seropositive rheumatoid arthritis (RA+), ankylosing spondylitis (AS), ulcerative colitis (UC), Crohn's disease (CD), systemic lupus erythematosus (SLE).

Disease	Glycan	Effect	Standard error	*p*-value	Adjusted *p*-value
**CD**	**G0F**	**0.95**	0.10	2.08E-20	**2.50E-19**
**CD**	**G1F**	**-0.69**	0.11	2.20E-10	**5.85E-10**
**CD**	**G2F**	**-0.99**	0.09	2.48E-23	**5.95E-22**
**CD**	**G2FS**	**-0.86**	0.10	2.82E-17	**2.25E-16**
**PsA**	**G0F**	**0.36**	0.10	2.09E-04	**2.79E-04**
**PsA**	**G1F**	**-0.41**	0.10	1.07E-04	**1.45E-04**
**PsA**	**G2F**	**-0.51**	0.09	1.05E-08	**2.29E-08**
**PsA**	**G2FS**	**-0.41**	0.09	8.17E-06	**1.20E-05**
**RA-**	**G0F**	**0.42**	0.15	4.40E-03	**5.28E-03**
**RA-**	**G1F**	**-0.38**	0.17	2.49E-02	**2.72E-02**
**RA-**	**G2F**	**-0.60**	0.13	6.29E-06	**9.64E-06**
**RA-**	**G2FS**	**-0.34**	0.14	1.67E-02	**1.85E-02**
**RA+**	**G0F**	**0.55**	0.12	3.32E-06	**5.32E-06**
**RA+**	**G1F**	**-0.67**	0.13	5.19E-07	**8.70E-07**
**RA+**	**G2F**	**-0.76**	0.10	5.82E-13	**2.46E-12**
**RA+**	**G2FS**	**-0.54**	0.12	4.30E-06	**6.73E-06**
**SLE**	**G0F**	**0.41**	0.13	1.34E-03	**1.73E-03**
**SLE**	**G1F**	**-0.75**	0.14	1.31E-07	**2.42E-07**
**SLE**	**G2F**	**-0.86**	0.12	1.90E-12	**6.84E-12**
**SLE**	**G2FS**	**-0.60**	0.12	1.67E-06	**2.74E-06**
**UC**	**G0F**	**0.61**	0.11	2.73E-08	**5.78E-08**
**UC**	**G1F**	**-0.85**	0.11	2.44E-13	**1.17E-12**
**UC**	**G2F**	**-0.79**	0.10	1.09E-13	**5.63E-13**
**UC**	**G2FS**	**-0.58**	0.11	8.39E-08	**1.59E-07**

Table S4: Baseline associations of IgG4 Fc glycans with various chronic inflammatory diseases from the Cohort 1. *p*-values were adjusted for multiple testing and considered significant if <0.05 (bold). Effect: the difference of a glycan trait (expressed in standard deviation units) between patient and control groups. Diseases: psoriatic arthritis (PsA), seronegative rheumatoid arthritis (RA-), seropositive rheumatoid arthritis (RA+), ankylosing spondylitis (AS), ulcerative colitis (UC), Crohn's disease (CD), systemic lupus erythematosus (SLE).

Disease	Glycan	Effect	Standard error	*p*-value	Adjusted *p*-value
**CD**	**G0F**	0.11	0.11	2.97E-01	3.10E-01
**CD**	**G1F**	**-0.73**	0.10	6.29E-13	**2.52E-12**
**CD**	**G2F**	**-1.03**	0.10	9.75E-23	**1.76E-21**
**CD**	**G2FS**	**-0.96**	0.10	1.67E-19	**1.71E-18**
**PsA**	**G0F**	**0.32**	0.10	2.05E-03	**2.59E-03**
**PsA**	**G1F**	**-0.27**	0.11	1.19E-02	**1.38E-02**
**PsA**	**G2F**	**-0.56**	0.10	7.31E-08	**1.42E-07**
**PsA**	**G2FS**	**-0.53**	0.10	3.42E-07	**6.01E-07**
**RA-**	**G0F**	**0.40**	0.17	1.49E-02	**1.70E-02**
**RA-**	**G1F**	-0.12	0.17	4.67E-01	4.80E-01
**RA-**	**G2F**	**-0.55**	0.16	9.26E-04	**1.21E-03**
**RA-**	**G2FS**	**-0.47**	0.17	5.88E-03	**6.94E-03**
**RA+**	**G0F**	**0.32**	0.13	1.53E-02	**1.72E-02**
**RA+**	**G1F**	**-0.40**	0.14	3.19E-03	**3.90E-03**
**RA+**	**G2F**	**-0.83**	0.13	1.86E-10	**5.15E-10**
**RA+**	**G2FS**	**-0.61**	0.13	7.64E-06	**1.15E-05**
**SLE**	**G0F**	-0.04	0.14	7.49E-01	7.59E-01
**SLE**	**G1F**	**-0.57**	0.14	3.46E-05	**4.88E-05**
**SLE**	**G2F**	**-0.98**	0.13	2.19E-12	**7.51E-12**
**SLE**	**G2FS**	**-0.87**	0.14	9.23E-10	**2.14E-09**
**UC**	**G0F**	**0.34**	0.11	2.09E-03	**2.59E-03**
**UC**	**G1F**	**-0.60**	0.11	6.69E-08	**1.34E-07**
**UC**	**G2F**	**-0.85**	0.11	3.11E-13	**1.40E-12**
**UC**	**G2FS**	**-0.61**	0.12	2.22E-07	**4.00E-07**

Table S5: Changes in IgG Fc glycan profiles during therapy in three cohorts (Cohort 1 - 26 weeks, Cohort 2- 30 weeks, and Cohort 3 - 14 weeks of therapy) of UC and CD patients. Effect: weekly change in glycan trait (expressed in standard deviation units). *p*-values were adjusted for multiple testing and considered significant if <0.05 (bold). Changes in IgG1 Fc glycan patterns during treatment for individual cohorts are visualised in Error! Reference source not found. **and the results of the meta-analysis of all three cohorts are shown in** Error! Reference source not found.**.**

		Cohort 1			Cohort 2			Cohort 3		
IgG subclass	Glycan	Effect	Standard error	*p*-value	Effect	Standard error	*p*-value	Effect	Standard error	*p*-value
IgG1	G0F	**-3.56E-02**	6.13E-03	**1.34E-06**	**-2.53E-02**	6.37E-03	**1.77E-04**	**-2.33E-02**	9.44E-03	**1.97E-02**
	G1F	2.89E-03	4.88E-03	5.54E-01	-2.20E-03	5.59E-03	6.94E-01	1.48E-03	9.19E-03	8.72E-01
	G2F	**2.83E-02**	5.04E-03	**2.45E-06**	**1.59E-02**	4.89E-03	**1.89E-03**	**2.47E-02**	6.83E-03	**1.14E-03**
	G2FS1	**4.03E-02**	5.06E-03	**3.65E-09**	**1.73E-02**	4.74E-03	**5.31E-04**	**3.48E-02**	9.03E-03	**6.49E-04**
IgG2/3	G0F	**-2.77E-02**	4.51E-03	**6.99E-07**	**-1.29E-02**	4.72E-03	**7.95E-03**	**-2.33E-02**	8.91E-03	**1.56E-02**
	G1F	5.72E-03	5.18E-03	2.74E-01	5.14E-03	4.94E-03	2.99E-01	-3.33E-03	9.10E-03	7.15E-01
	G2F	**2.48E-02**	3.69E-03	**1.06E-07**	**1.79E-02**	4.19E-03	**6.52E-05**	**1.57E-02**	6.63E-03	**2.41E-02**
	G2FS1	**2.95E-02**	3.49E-03	**6.14E-10**	**1.26E-02**	3.89E-03	**1.95E-03**	**1.81E-02**	8.33E-03	**4.03E-02**
IgG4	G0F	**-3.04E-02**	6.38E-03	**4.52E-05**	**-1.53E-02**	4.13E-03	**3.55E-04**	**-1.84E-02**	7.30E-03	**1.82E-02**
	G1F	-8.02E-03	4.53E-03	8.84E-02	-5.24E-03	3.74E-03	1.66E-01	-6.83E-03	7.28E-03	3.55E-01
	G2F	**1.49E-02**	5.90E-03	**1.63E-02**	7.30E-03	5.66E-03	2.05E-01	1.52E-02	7.55E-03	5.12E-02
	G2FS1	**2.50E-02**	5.54E-03	**6.98E-05**	5.75E-03	4.69E-03	2.22E-01	**1.78E-02**	6.34E-03	**8.64E-03**

Table S6: Changes in IgG1 Fc glycans during 26 weeks on therapy in the Cohort 1. Analysis was performed by implementing a linear mixed-effects model, with time as a fixed effect and the individual sample measurement as a random effect. *p*-values were adjusted for multiple testing and considered significant if <0.05 (bold). Effect: weekly change in glycan trait (expressed in standard deviation units). Diseases: ankylosing spondylitis (AS), Crohn's disease (CD), psoriatic arthritis (PsA), seronegative rheumatoid arthritis (RA-), seropositive rheumatoid arthritis (RA+), ulcerative colitis (UC).

Glycan name	Disease	Effect	Standard error	*p*-value	Adjusted *p*-value
**G0F**	**AS**	**-2,59E-02**	6,02E-03	6,52E-04	**4,64E-03**
**G1F**	**AS**	-1,21E-03	6,36E-03	8,50E-01	8,81E-01
**G2F**	**AS**	**1,52E-02**	4,72E-03	4,36E-03	**1,26E-02**
**G2FS**	**AS**	**2,34E-02**	6,94E-03	3,72E-03	**1,16E-02**
**G0F**	**CD**	**-1,81E-02**	4,31E-03	2,64E-04	**3,17E-03**
**G1F**	**CD**	1,14E-02	7,13E-03	1,15E-01	1,97E-01
**G2F**	**CD**	**2,37E-02**	6,49E-03	9,32E-04	**4,79E-03**
**G2FS**	**CD**	**2,63E-02**	6,84E-03	5,03E-04	**4,64E-03**
**G0F**	**PsA**	-1,66E-02	8,15E-03	4,71E-02	9,17E-02
**G1F**	**PsA**	9,68E-03	6,80E-03	1,66E-01	2,57E-01
**G2F**	**PsA**	1,95E-02	8,94E-03	3,29E-02	6,98E-02
**G2FS**	**PsA**	**1,88E-02**	5,82E-03	2,82E-03	**1,01E-02**
**G0F**	**RA-**	**-2,17E-02**	6,93E-03	5,23E-03	**1,45E-02**
**G1F**	**RA-**	8,23E-03	6,18E-03	1,93E-01	2,83E-01
**G2F**	**RA-**	**2,26E-02**	7,30E-03	6,45E-03	**1,72E-02**
**G2FS**	**RA-**	**2,01E-02**	7,04E-03	1,24E-02	**2,99E-02**
**G0F**	**RA+**	**-2,05E-02**	4,93E-03	1,83E-04	**3,17E-03**
**G1F**	**RA+**	4,34E-03	5,61E-03	4,46E-01	5,45E-01
**G2F**	**RA+**	**1,54E-02**	3,77E-03	2,36E-04	**3,17E-03**
**G2FS**	**RA+**	**2,26E-02**	4,88E-03	4,74E-05	**3,00E-03**
**G0F**	**UC**	-4,88E-03	5,91E-03	4,18E-01	5,28E-01
**G1F**	**UC**	-4,77E-03	7,26E-03	5,16E-01	6,19E-01
**G2F**	**UC**	1,28E-03	8,98E-03	8,87E-01	9,00E-01
**G2FS**	**UC**	2,66E-03	8,66E-03	7,59E-01	8,10E-01

Table S7: Changes in IgG2/3 Fc glycans during 26 weeks on therapy in the Cohort 1. Analysis was performed by implementing a linear mixed-effects model, with time as a fixed effect and the individual sample measurement as a random effect. *p*-values were adjusted for multiple testing and considered significant if <0.05 (bold). Effect: weekly change in glycan trait (expressed in standard deviation units). Diseases: ankylosing spondylitis (AS), Crohn's disease (CD), psoriatic arthritis (PsA), seronegative rheumatoid arthritis (RA-), seropositive rheumatoid arthritis (RA+), ulcerative colitis (UC).

Glycan name	Disease	Effect	Standard error	*p*-value	Adjusted *p*-value
**G0F**	**AS**	**-2,56E-02**	4,92E-03	8,33E-05	**3,00E-03**
**G1F**	**AS**	-5,89E-03	6,17E-03	3,42E-01	4,56E-01
**G2F**	**AS**	**1,52E-02**	3,51E-03	1,33E-03	**6,40E-03**
**G2FS**	**AS**	**1,81E-02**	4,80E-03	3,16E-03	**1,08E-02**
**G0F**	**CD**	**-1,19E-02**	3,58E-03	2,10E-03	**8,63E-03**
**G1F**	**CD**	1,99E-03	5,16E-03	6,99E-01	7,78E-01
**G2F**	**CD**	**1,45E-02**	4,39E-03	2,16E-03	**8,63E-03**
**G2FS**	**CD**	**1,92E-02**	5,00E-03	7,21E-04	**4,64E-03**
**G0F**	**PsA**	-1,17E-02	9,28E-03	2,40E-01	3,38E-01
**G1F**	**PsA**	3,31E-03	7,00E-03	6,37E-01	7,40E-01
**G2F**	**PsA**	**3,61E-02**	1,31E-02	8,10E-03	**2,08E-02**
**G2FS**	**PsA**	1,95E-02	1,13E-02	1,05E-01	1,89E-01
**G0F**	**RA-**	**-1,76E-02**	5,18E-03	3,35E-03	**1,09E-02**
**G1F**	**RA-**	6,16E-03	7,74E-03	4,27E-01	5,30E-01
**G2F**	**RA-**	**1,99E-02**	6,36E-03	4,28E-03	**1,26E-02**
**G2FS**	**RA-**	1,51E-02	6,73E-03	3,84E-02	7,89E-02
**G0F**	**RA+**	**-2,09E-02**	4,97E-03	2,53E-04	**3,17E-03**
**G1F**	**RA+**	-5,96E-03	6,35E-03	3,61E-01	4,73E-01
**G2F**	**RA+**	**1,59E-02**	4,67E-03	2,44E-03	**9,26E-03**
**G2FS**	**RA+**	**1,96E-02**	4,93E-03	7,51E-04	**4,64E-03**
**G0F**	**UC**	-2,58E-03	6,63E-03	7,02E-01	7,78E-01
**G1F**	**UC**	-2,71E-03	7,64E-03	7,46E-01	8,10E-01
**G2F**	**UC**	-3,70E-04	5,84E-03	9,51E-01	9,51E-01
**G2FS**	**UC**	1,19E-02	8,63E-03	1,72E-01	2,59E-01

Table S8: Changes in IgG4 Fc glycans during 26 weeks on therapy in the Cohort 1. Analysis was performed by implementing a linear mixed-effects model, with time as a fixed effect and the individual sample measurement as a random effect. *p*-values were adjusted for multiple testing and considered significant if <0.05 (bold). Effect: weekly change in glycan trait (expressed in standard deviation units). Diseases: ankylosing spondylitis (AS), Crohn's disease (CD), psoriatic arthritis (PsA), seronegative rheumatoid arthritis (RA-), seropositive rheumatoid arthritis (RA+), ulcerative colitis (UC).

Glycan name	Disease	Effect	Standard error	Time *p*-value	Adjusted *p*-value
**G0F**	**AS**	**-1,18E-02**	4,36E-03	2,29E-02	**5,00E-02**
**G1F**	**AS**	-6,06E-03	3,45E-03	1,21E-01	1,98E-01
**G2F**	**AS**	9,46E-03	6,12E-03	1,68E-01	2,57E-01
**G2FS**	**AS**	1,07E-02	5,91E-03	1,13E-01	1,97E-01
**G0F**	**CD**	**-1,74E-02**	4,74E-03	7,73E-04	**4,64E-03**
**G1F**	**CD**	-5,37E-03	4,61E-03	2,54E-01	3,51E-01
**G2F**	**CD**	1,09E-02	6,35E-03	9,67E-02	1,79E-01
**G2FS**	**CD**	**1,69E-02**	6,19E-03	1,15E-02	**2,86E-02**
**G0F**	**PsA**	-1,67E-02	7,96E-03	4,38E-02	8,75E-02
**G1F**	**PsA**	-1,23E-03	6,78E-03	8,57E-01	8,81E-01
**G2F**	**PsA**	1,79E-02	1,03E-02	9,00E-02	1,71E-01
**G2FS**	**PsA**	7,96E-03	6,15E-03	2,03E-01	2,93E-01
**G0F**	**RA-**	**-1,22E-02**	4,74E-03	1,64E-02	**3,69E-02**
**G1F**	**RA-**	7,84E-03	4,82E-03	1,29E-01	2,06E-01
**G2F**	**RA-**	**2,25E-02**	7,96E-03	1,42E-02	**3,31E-02**
**G2FS**	**RA-**	1,44E-02	8,76E-03	1,21E-01	1,98E-01
**G0F**	**RA+**	**-1,78E-02**	4,86E-03	6,14E-04	**4,64E-03**
**G1F**	**RA+**	2,64E-03	4,23E-03	5,42E-01	6,39E-01
**G2F**	**RA+**	**1,82E-02**	4,96E-03	8,95E-04	**4,79E-03**
**G2FS**	**RA+**	**2,03E-02**	5,70E-03	1,67E-03	**7,49E-03**
**G0F**	**UC**	2,40E-03	7,26E-03	7,65E-01	8,10E-01
**G1F**	**UC**	5,65E-03	6,68E-03	4,01E-01	5,16E-01
**G2F**	**UC**	-5,25E-03	1,14E-02	6,61E-01	7,55E-01
**G2FS**	**UC**	1,55E-02	1,34E-02	2,58E-01	3,51E-01

Table S9: Associations between the rate of change of IgG Fc glycan profiles and disease activity during UC and CD treatment in three cohorts (Cohort 1 - 26 weeks, Cohort 2 - 30 weeks, and Cohort 3 - 14 weeks). Effect: the difference of a glycan trait (expressed in standard deviation units) during treatment between remission and non-remission groups of patients. *p*-values <0.05 were considered significant (bold). Changes in IgG glycan patterns with time on therapy in patients with active vs. inactive disease are visualized in Figure 3, Figure S1 and Figure S2, while the meta-analysis of all three cohorts is shown in Error! Reference source not found.**.**

		**Cohort 1**			**Cohort 2**			**Cohort 3**		
**IgG subclass**	**Glycan**	**Effect**	**Standard error**	***p*-value**	**Effect**	**Standard error**	***p*-value**	**Effect**	**Standard error**	***p*-value**
**IgG1**	**G0F**	-1.25E-02	1.34E-02	3.64E-01	-1.02E-02	1.10E-02	3.62E-01	-2.84E-02	1.71E-02	1.07E-01
	**G1F**	-5.50E-04	9.89E-03	9.56E-01	-9.45E-03	9.72E-03	3.34E-01	4.92E-03	1.86E-02	7.94E-01
	**G2F**	3.15E-03	1.02E-02	7.69E-01	7.64E-03	9.72E-03	4.37E-01	1.83E-02	1.35E-02	1.79E-01
	**G2FS1**	1.21E-02	9.75E-03	3.04E-01	4.11E-03	9.50E-03	6.65E-01	7.26E-03	1.84E-02	6.93E-01
**IgG2/3**	**G0F**	-1.21E-02	8.16E-03	1.69E-01	-6.91E-03	8.68E-03	4.29E-01	**-4.22E-02**	1.61E-02	**1.27E-02**
	**G1F**	5.07E-03	9.41E-03	5.91E-01	1.63E-02	8.50E-03	5.79E-02	1.81E-02	1.40E-02	2.03E-01
	**G2F**	1.48E-02	8.02E-03	7.66E-02	**1.65E-02**	8.13E-03	**4.81E-02**	**2.91E-02**	1.21E-02	**2.24E-02**
	**G2FS1**	1.28E-03	8.07E-03	8.77E-01	7.41E-03	8.08E-03	3.63E-01	**3.45E-02**	1.51E-02	**2.72E-02**
**IgG4**	**G0F**	-2.00E-02	1.40E-02	1.69E-01	-1.39E-02	7.35E-03	6.32E-02	-2.44E-02	1.33E-02	7.32E-02
	**G1F**	-8.89E-03	1.12E-02	4.49E-01	-9.70E-04	7.65E-03	9.01E-01	-1.56E-02	1.39E-02	2.67E-01
	**G2F**	-9.65E-03	1.36E-02	4.91E-01	4.10E-03	1.09E-02	7.09E-01	6.90E-03	1.52E-02	6.53E-01
	**G2FS1**	3.98E-03	1.34E-02	7.88E-01	5.46E-03	9.47E-03	5.65E-01	1.27E-02	1.27E-02	3.20E-01

Table S10: Baseline associations of IgG Fc glycans with future disease activity, i.e. response to therapy in three cohorts (Cohort 1 - 26 weeks, Cohort 2 - 30 weeks, and Cohort 3 - 14 weeks on therapy) of UC and CD patients. *p*-values were adjusted for multiple testing and were considered significant if <0.05 (bold). Effect: baseline difference of a glycan trait (expressed in standard deviation units) between remission and non-remission patients. *p*-values were adjusted for multiple testing and considered significant if <0.05 (bold).

IgG subclass	Glycan	Cohort 1			Cohort 2			Cohort 3			Meta-analysis			
		Effect	Standard error	*p*-value	Effect	Standard error	p value	Effect	Standard error	*p*-value	Effect	Standard error	*p*-value	Adjusted p value
IgG1	G0F	-9,97E-01	4,31E-01	1,78E-02	-2,02E-01	2,67E-01	4,40E-01	2,18E-01	4,56E-01	6,06E-01	-2,95E-01	2,03E-01	1,46E-01	9,07E-01
	G1F	-3,18E-01	5,31E-01	5,22E-01	2,20E-01	2,54E-01	3,76E-01	5,09E-01	4,22E-01	1,99E-01	2,09E-01	2,01E-01	3,00E-01	9,07E-01
	G2F	-1,05E+00	4,78E-01	2,41E-02	-2,58E-01	2,66E-01	3,22E-01	6,00E-01	3,91E-01	1,05E-01	-1,72E-01	2,00E-01	3,90E-01	9,07E-01
	G2FS1	-1,28E+00	4,80E-01	7,12E-03	-1,05E-01	2,68E-01	6,89E-01	5,94E-01	4,24E-01	1,37E-01	-1,55E-01	2,05E-01	4,49E-01	9,07E-01
IgG2/3	G0F	-1,23E+00	4,27E-01	3,98E-03	1,99E-01	2,63E-01	4,40E-01	1,44E-01	4,60E-01	7,35E-01	-1,29E-01	2,01E-01	5,22E-01	9,07E-01
	G1F	-2,51E-01	4,87E-01	5,81E-01	1,12E-01	2,59E-01	6,58E-01	4,16E-01	4,23E-01	2,93E-01	1,19E-01	2,01E-01	5,55E-01	9,07E-01
	G2F	-6,78E-01	4,74E-01	1,32E-01	3,56E-01	2,56E-01	1,58E-01	3,51E-02	4,32E-01	9,30E-01	1,04E-01	2,00E-01	6,03E-01	9,07E-01
	G2FS1	3,17E-02	5,35E-01	9,49E-01	2,32E-01	2,51E-01	3,47E-01	-2,22E-01	4,08E-01	5,58E-01	9,67E-02	1,99E-01	6,27E-01	9,07E-01
IgG4	G0F	1,80E-01	5,14E-01	7,07E-01	-1,06E-01	2,62E-01	6,79E-01	-2,19E-01	4,38E-01	5,91E-01	-8,50E-02	2,06E-01	6,80E-01	9,07E-01
	G1F	4,65E-01	4,89E-01	3,11E-01	-3,80E-02	2,68E-01	8,85E-01	-5,76E-02	4,55E-01	8,91E-01	4,98E-02	2,09E-01	8,12E-01	9,74E-01
	G2F	-2,43E-01	4,81E-01	5,88E-01	1,25E-02	2,62E-01	9,61E-01	2,66E-01	4,47E-01	5,22E-01	1,95E-02	2,05E-01	9,24E-01	9,79E-01
	G2FS1	-7,21E-01	5,24E-01	1,47E-01	-2,42E-01	2,67E-01	3,53E-01	9,01E-01	3,91E-01	1,79E-02	-5,31E-03	2,03E-01	9,79E-01	9,79E-01

Table S11: Description of patient cohorts analyzed in the study. F - female; M - male, n.a. - not available, CD - number of Crohn's disease patients in the cohort; UC – number of UC patients in the cohort; CD/UC – number of patients not differentially diagnosed as either UC or CD. Seropositive rheumatoid arthritis - RA+, seronegative rheumatoid arthritis - RA-, psoriatic arthritis - PsA, ankylosing spondylitis - AS, systemic lupus erythematosus - SLE.

Cohort	Patients	F	M	n. a.	Age (years)	CD	UC	RA +	RA -	PsA	AS	SLE	Therapy	Duration of follow-up (weeks)
**Cohort 3**	42	25	17	0	16-74	20	22	0	0	0	0	0	infliximab, vedolizumab,	14
**Cohort 1**	145	93	53	0	18-67	32	12	37	19	22	17	6	infliximab	104
**Cohort 2**	61	n.a.	n.a.	n.a.	18-80	61	0	0	0	0	0	0	anti-TNF (36), azathioprine (25)	30
**Controls**	204	88	115	1	27-74	0	0	0	0	0	0	0	non-CID therapy	n.a.

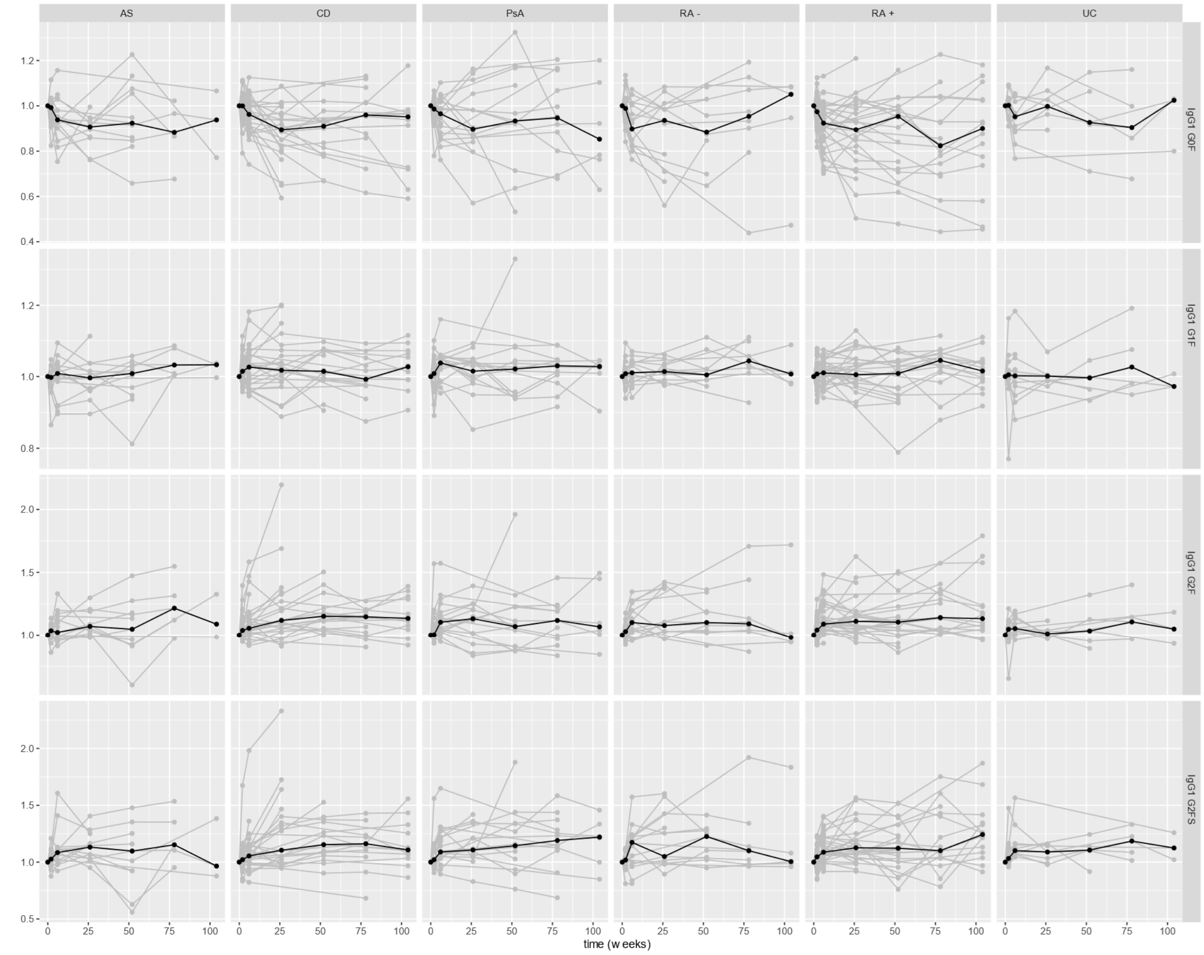

Figure S1: Changes in IgG1 Fc glycan profiles during treatment in six inflammatory diseases from the Cohort 1. Ankylosing spondylitis (AS), Ulcerative colitis (UC), Crohn's disease (CD), psoriatic arthritis (PsA), seronegative rheumatoid arthritis (RA-), and seropositive rheumatoid arthritis (RA+). Median glycan values for each time point are shown in bold. Y-axis: relative change in glycan value normalized to baseline (1st timepoint); X-axis: duration of follow-up (weeks).

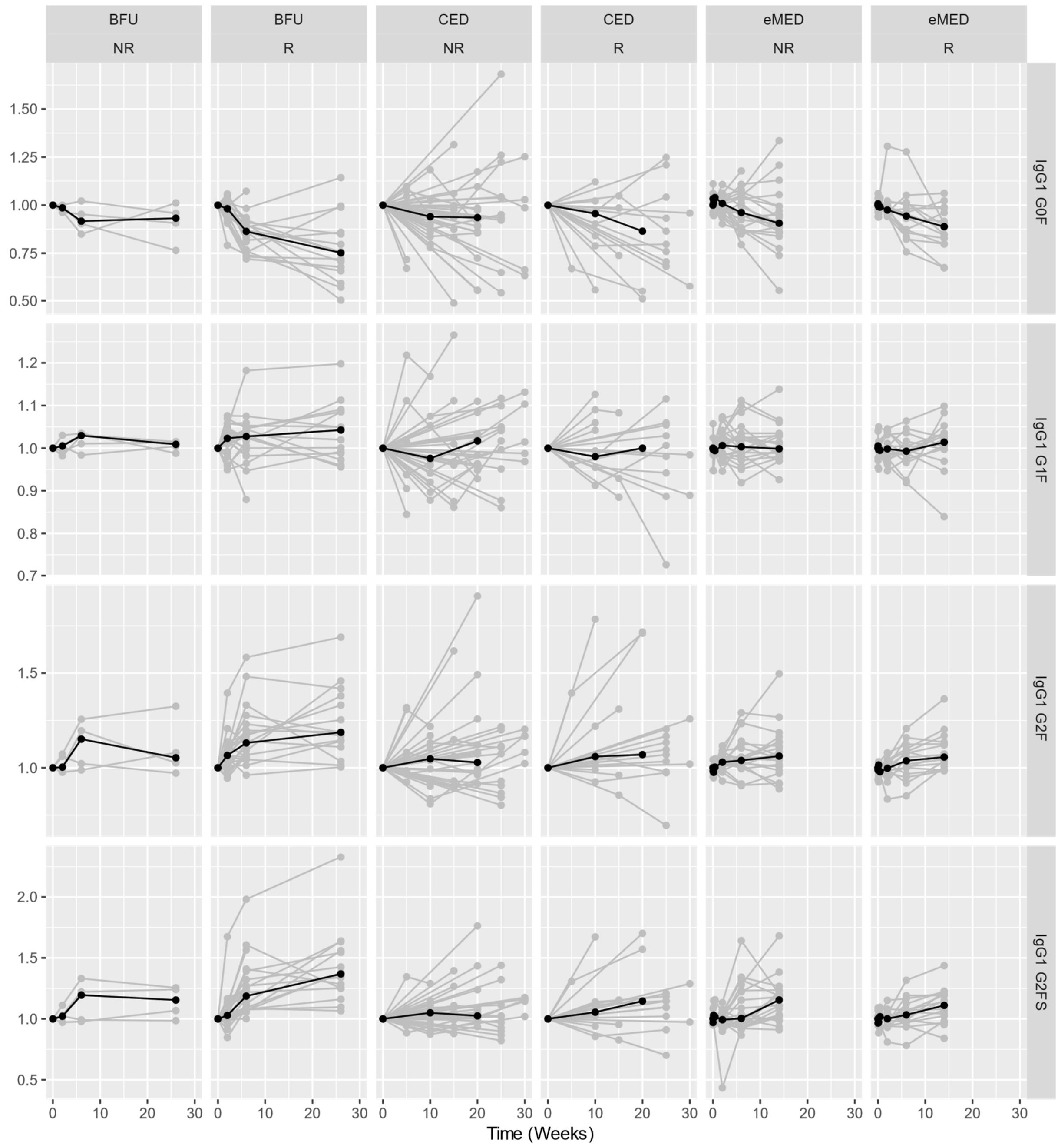

Figure S2: Changes in IgG1 Fc glycan profiles during treatment in patients that entered remission (R) vs. patients with active disease (NR) in three cohorts (Cohort 1, Cohort 2, Cohort 3) of UC and CD patients. Median glycan values for each time point are bolded. Y-axis: relative change in glycan value normalized to baseline (1st timepoint); X-axis: duration of follow-up (weeks). Associations of IgG Fc glycan profiles with disease activity during UC and CD treatment in Cohort 1, Cohort 2, and Cohort 3 are shown in Table S9 while the results of meta-analysis of all three cohorts are presented in Error! Reference source not found.**.**

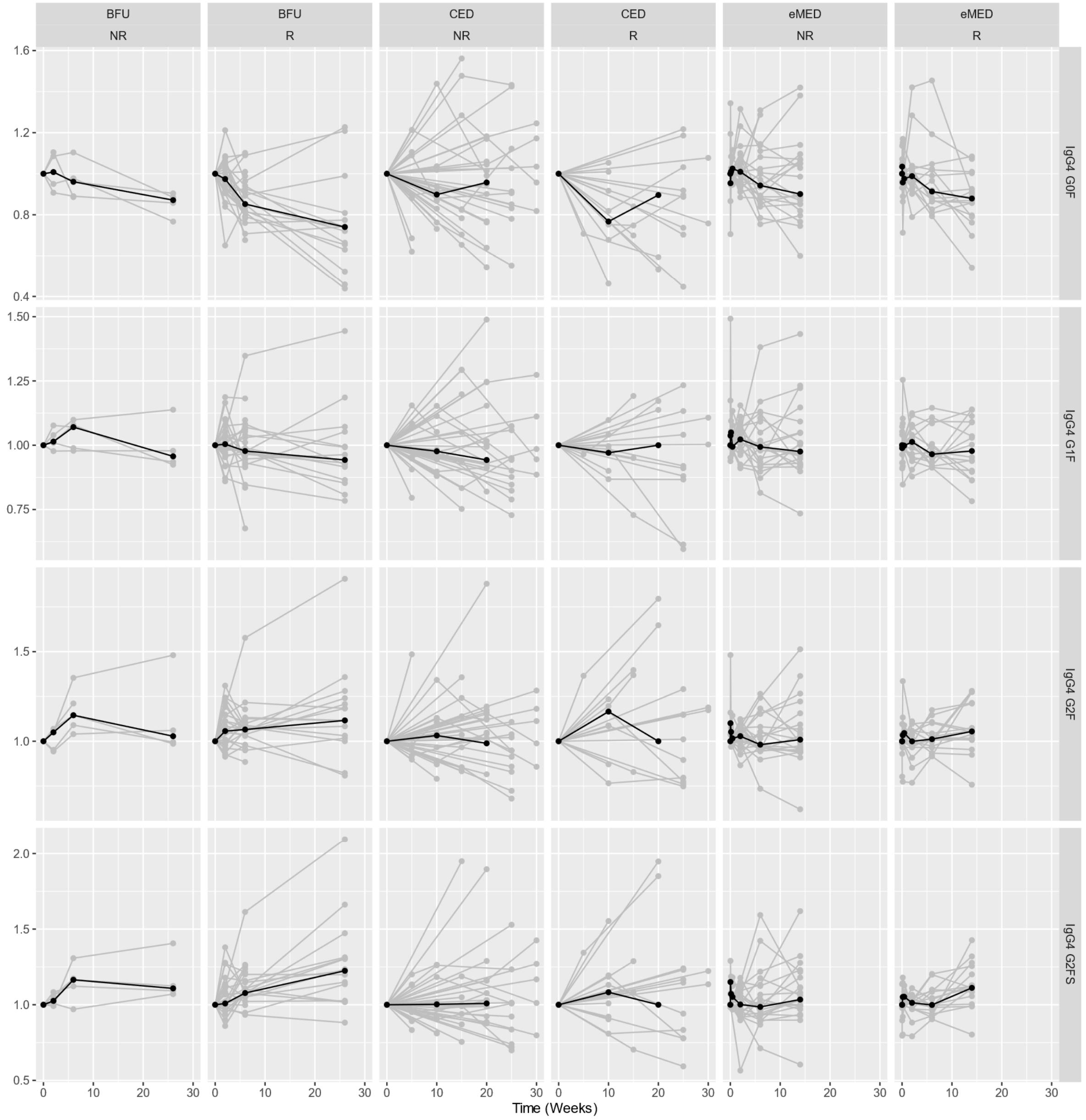

Figure S3: Changes in IgG4 Fc glycan profiles during treatment in patients that entered remission (R) vs. patients with active disease (NR) in three cohorts of UC and CD patients. Median glycan values for each time point are bolded. Y-axis: relative change in glycan value normalized to baseline (1st timepoint); X-axis: duration of follow-up (weeks). Associations of IgG Fc glycan profiles with disease activity during treatment in Cohort 1, Cohort 2, and Cohort 3 are shown in Table S9 while the results of meta-analysis of all three cohorts are presented in Error! Reference source not found.**.**
